# Fabrication of nano‐encapsulated angelica (*Heracleum persicum*) essential oil for enriching dairy dessert: Physicochemical, rheological and sensorial properties

**DOI:** 10.1049/nbt2.12112

**Published:** 2023-01-27

**Authors:** Narin Mhemmedamin Nanakali

**Affiliations:** ^1^ Department of Food Science and Technology College of Agriculture Engineering Sciences Salahaddin Univeristy‐Erbil Erbil Iraq

**Keywords:** antibacterial activity, dairy dessert, encapsulation, essential oils, free radicals, nanobiotechnology

## Abstract

In this study, the nanoemulsions containing angelica essential oil (AEO) was used as a novel nano‐carrier for enrichment of dairy dessert. Firstly, oil‐in‐water nanoemulsions were prepared by different levels of GE (1%, 5%, 10%, and 15%) as the dispersed phase, Tween 80 as surfactant with a constant surfactant to essential oil ratio (1:1), and distillated water as a continuous phase. Droplet size, free radical scavenging capacity, antimicrobial activity against gram‐positive (*Staphylococcus aureus* (*25923 ATCC*)) and gram‐negative (*Escherichia coli H7 O157 (700728 ATCC*)) were evaluated for produced nanoemulsions. The mean droplet size of nanoemulsion increased from 75 to 95 nm and antioxidant capacity also enhanced from 15.4% to 30.2% by increasing AEO level from 1% to 15%. Antimicrobial analysis by disk diffusion methods for nanoemulsions containing different levels of AEO cleared that nanoemulsions with high levels of AEO showed the stronger antimicrobial activity against both used bacteria and especially more activity against *Staphylococcus aureus*. The results of the total count and yeast and mould count show that the nanoemulsions with different levels of AEO have been effective on the number of microorganisms, particularly during storage. The incorporation of pure essential oil and nanoemulsions with different levels of AEO did not affect significantly the pH of different dessert samples however, they affected the dry matter and free radical scavenging capacity. Adding of nanoemulsions with different levels of AEO to the desserts had a considerable effect on the rheological properties including apparent viscosity, Gʹ, G", Tan *δ* and complex viscosity and all samples showed shear‐thining behaviour. Results from organoleptic characteristics (taste, odour colour, mouthfeel and total acceptance) showed that enriched samples by nanoemulsions, particularly with higher level of AEO had higher sensorial scores. In general, samples containing free AEO (not encapsulated) had the lower scores in all organoleptic characteristics.

## Graphical abstract

In this project, an attempt has been made to enrich a children's favourite dairy product by nano‐encapsulatio of an antimicrobial and antioxidant compound (angelica essential oil) with a customer‐friendly scent compatible with the product. Such researches have grown day by day and the results were promising in this regard and highlighted the importance of nanotechnology in this field.

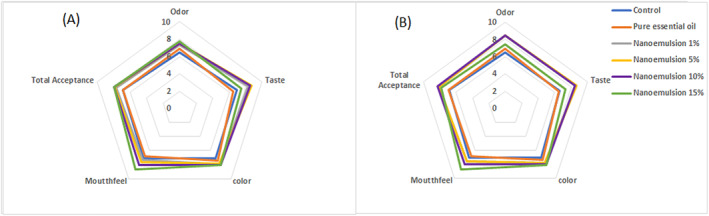

## 1 INTRODUCTION

*Heracleum persicum*, a flowering plant of the family *Apiaceae*, naturally grows under humid conditions and has traditionally been used as a medicinal herb and flavouring agent [1]. *Heracleum persicum* has been extensively used as food additives, food preservatives, flavouring agents and spices and for the treatment purposes of gastrointestinal, neurological, respiratory, urinary and rheumatologically dysfunctions [2]. Functional properties of *H*. *persicum* includes antidiabetic, antihyperlipidemic, antioxidant, antimicrobial, anti‐inflammatory, anticonvulsant, analgesic and cardio‐ and gastro‐protective properties [3, 4]. The main bioactive components of this plant are volatiles, ethyl esters, n‐alkenes, phenolics, flavonoids, alkaloids, terpenoids and triterpenes [2]. Also, Hexyl butyrate, anethole, octyl acetate, hexyl‐2‐methylbutanoate and hexyl isobutyrate were found to be the main natural phytochemicals of *H*. *persicum* essential oil [5].

Essential oils are natural aromatic compounds found in the roots, rhizomes, wood bark, leaves, stems, fruit, flowers and seeds, and other parts of plants. Essential oils contain many complex chemical compounds having anti‐inflammatory, antibacterial, antimicrobial, and antiviral properties. Essential oils are highly concentrated and a small amount is very potent and consists of esters, aldehydes, ketones, and alcohols. Preserving milk by adding essential oils is a nontraditional technique in order to satisfy customers need, and the chapter deals in details with application of essential oils in milk and milk products and the different techniques used for masking the strong odour of the oils [6].

Nanoemulsions are the emulsions having droplet size below 100 nm developed by adopting pressure‐ or energy‐based methods like homogenizers with a high‐pressure valve or using microfluidisers [7]. Within the droplets, the combination of functional food components is possible with the continuous or interfacial region. It provides the ability to encapsulate and release at a single delivering system. Such systems can carry numerous functional components, and the release of these components is controlled with a particular environmental trigger [8].

In milk and milk‐based products, various organoleptic characteristics like mouthfeel, taste, flavour, consistency, and rheological characteristics are considered as the quality determinants. The achievement of desired quality parameters in a product can be done by controlling the distribution and droplet size assisted by emulsification process. The stabilising ability of emulsion in milk is possible without using any extraneous stabilising agents due to inherent emulsifying capacity of milk proteins. The demand for nanoemulsions over conventional emulsions is increasing day by day, and research is emphasised on their particular applications and properties like functional beverages and foods, improved bioavailability of nutrients, and enhanced physical stability. The dairy‐based products can be used as ingredients which are having different functions that supply physical stability as well as health and nutritional benefits [7].

There are numerous types of dairy desserts which are mainly formulated with milk, cream and butter, thickeners (e.g., starch and hydrocolloids), sucrose, colourants and flavouring agents. They are consumed either with a meal or as a nutritious snack [9]. Dairy desserts are good sources of energy and calcium and hence can promote bone health, reduce the risk of chronic diseases and improve overall health [10]. However, the addition of functional components in their recipe can boost their nutritional value as well as consumer satisfaction. In recent years, increasing researches have been conducted on the use of nanoemulsions containing plant extracts and essential oils in the formulation of various food, drug and cosmetic products, which in the closest research, Bashlouei et al [5] prepared *Heracleum persicum* oil nanoemulsion and investigated biological properties against human breast cancer cells and normal human fibroblasts foreskin. They explained that *Heracleum persicum* oil nanoemulsion could be an eco‐friendly nanotherapeutic option for pharmaceutical, cosmetic and food applications. The present research is a continuation of the research of other authors which focused more on the food applications of this nanoemulsion and tried to investigate the effect of adding this type of nanoemulsion on the physicochemical, sensorial and rheological properties of a widely consumed dairy product (especially in children).

## 2 MATERIAL AND METHODS

### 2.1 Materials

The dried angelica plant was purchased from the market. Methanol (96%) and sodium hydroxide were purchased from Merck Co and also PCA, Tween 80 and DPPH were prepared from Sigma‐Aldrich Co.

### 2.2 Preparation of essential oil

Dried leaves of angelica in room temperature, were separated from the other parts of plant and subjected to the hydro distillation for 3 h using a glass Clevenger type apparatus, according to the method recommended by the European Pharmacopoeia. The obtained essential oil was stored in sterilised dark glass at 4°C until experiments [11].

### 2.3 Preparation of nanoemulsions

Essential oil of angelica and tween 80 were mixed by magnetic stirrer for 30 min at 500 rpm and then the mixed oil phase (tween 80 and essential oil of angelica mixture) was added slowly to the aqueous phase while mixing by magnetic stirrer for 30 min at 700 rpm (25°C ± 3°C). Then, the produced premixed emulsions were transferred to the water bath ultrasonic with 100 w powers and 40 kHz frequency for 15 min (30°C ± 5°C). The ratio of surfactant‐to‐oil was fixed for all of the nanoemulsions (1:1), while the aqueous phase content was varied (50%, 70%, 80%, and 90%) [12].

### 2.4 Droplet size distribution analysis

To measure particles, all of nanoemulsions are diluted 1: 50 in oil to prevent multiple particle scattering. The mean particle size (Z_average_) and polydispersity Index (PDI) were determined by dynamic light scattering (Malvern instrument, U.K) at the temperature of 25°C and an angle of 90° [13].

### 2.5 Free radical scavenging capacity

The antioxidant properties of the prepared nanoemulsions, as well as garlic extract, were each measured separately by DPPH method. In this method, 4 ml of 60 μM free radical methanol solution of DPPH was mixed with 0.2 ml of the prepared nanoemulsion and kept at room temperature for 60 min. Then the absorbance of the solution at 517 nm was observed using a spectrophotometer. Also, 4 ml of DPPH with a sample containing 0.2 ml of methanol was determined as a control sample and radical scavenging capacity was calculated using the relevant formula (Equation (1)) [14, 15].

(1) $$Free\;radical\;scavenging=\frac{control\;absorbance-sample\;absorbance}{control\;absorbance}\times 100$$

### 2.6 Microbial test by disk diffusion

The studied bacteria including *Staphylococcus aureus* (25923 ATCC) and *Escherichia coli H7 O157* (700728 ATCC) were cultured on Mueller‐Hinton agar and incubated for 24 h at 37°C. Then the dilution of 0.5 McFarland was prepared from the colonies of this environment by saline that its absorption was equal to 0.08–0.1 at a wavelength of 620 nm (the number of bacteria in this amount of absorbance is equal to 1.5 × 10^8^ CFU/ml). The bacteria spread on the Mueller‐Hinton agar by sterile swab and then paper discs (which immersed in the prepared nanoemulsion for 1 h before) were placed on plates. Finally, the plates were incubated at 37°C and the diameter of inhibition zone was measured using a millimetre rule after 24 h [13].

### 2.7 Preparation of dairy dessert

Control dessert was made with 10% sugar, 2% gelatin and 88% low‐fat milk. To prepare the enriched samples, nanoemulsions with different levels of angelica essential oil (AEO) (1%, 5%, 10% and 15%) but by a fixed ratio (10%) to the dairy dessert were mixed with one‐third of the milk and refrigerated at 7 ± 2°C for 2 h for a complete hydration. The remaining milk in a sealed glass jar was heated up in a water bath to 53 ± 1°C, and then, gelatin powder and sugar were added gradually while mixing. The mixing and heating continued until it reached 65°C. After that, the mixture was blended for 5 min using a blender (Brina, Model BHB‐341). The milk was added to the mixture in a glass jar container, capped and heated in a water bath at 72°C for 30 min [9].

### 2.8 Determination of dry matter and pH of the desserts

In a pre‐weighed and dried aluminium dish, 5.00 g of dessert was weighed and dried in an electrical oven at 105°C until constant weight. The mass of the dried sample was obtained from the difference between the mass of the dish containing sample before and after drying. The moisture content of the dessert was calculated using Equation (2).

(2) $$Moisture\;content\;\left(\%\right)=\frac{(Mass\;of\;dish\;and\;dessert\;before\;drying-Mass\;of\;dish\;and\;dessert\;after\;drying}{Mass\;of\;dish\;and\;dessert\;after\;drying}\times 100$$

Dry matter was calculated by subtracting the moisture content from 100. The pH of the dessert was determined after removing of the desserts from the refrigerator at 7°C using a calibrated digital pH metre (Model SK‐632PH; Metrohm, Herisau, Switzerland) [9].

### 2.9 Determination of released water from the desserts (synaeresis)

To determine the released water, the method of Siamand et al. [16] was followed with some modifications. The samples were removed from the plastic cups (see ‘Preparation of dairy dessert’ section) and placed between two filter papers (Whatman No. 4) of known weight and compressed with a 500 g weight for 10 min at room temperature. The water released from the sample was expressed as the increasing weight of filter papers, which was determined gravimetrically using an analytical balance.

### 2.10 Steady and oscillatory shear rheological analysis

Flow behaviour and oscillatory tests were performed in a rheometer (Anton Paar Physica MCR300). Flow properties of the mayonnaise samples were determined at 20°C using a parallel stainless steel plate having a diameter of 20 mm, in the shear rate range of 0.1 to 100 s^−1^. To characterise the flow behaviour, the experimental data were fitted to a Power‐law equation (Equation (3)):

(3) $$\tau =K{\dot{\gamma }}^{n}$$

Where τ is the shear stress (Pa), γ is the shear rate (1/s), *K* is the consistency index (Pa.sn), and *n* is the flow behaviour index.

The dynamic oscillatory tests were performed over a frequency range of 0.1–50 Hz at a constant strain of 0.5% (within the linear viscoelasticity range that was previously established by the strain sweep tests). Data were collected and rheological parameters were calculated using a rheometer software programme. Storage modulus (Gʹ) and loss modulus (Gʹʹ) and tan (delta) versus frequency was measured for all the samples [12].

### 2.11 Sensory evaluation

Sensory evaluation was conducted on the mayonnaise samples after 1‐day storage at room temperature. The mixed 5‐point hedonic structured scale was used (scale, 1 = the least acceptable; 5 = the most acceptable). The appearance, texture, taste, odour, and overall acceptance were evaluated by 12 panelists. Some bread and a cup of water were provided between samples to clean the palate.

### 2.12 Statistical analysis

All of the experiments were carried out in triplicate at least for mean and standard deviation calculation. Completely randomized design performed by Minitab software (Version 17) and Tukey test was used for comparison of mean treatments (*α* = 0.05).

## 3 RESULTS AND DISCUSSION

### 3.1 Droplet size and polydispersity index (PDI)

The results related to the particle size show that, in general, the particle size for all produced nanoemulsions is below 100 nm, and with the increase in the concentration of essential oil (as the dispersed phase in the nanoemulsion), a slight increase in the particle size is observed. Also, PDI also reported below 0.2 for majority of produced nanoemulsions which it may be due to application of suitable sonication and high ratio of surfactant to dispersed phase (essential oil) (Table 1). The droplet size of 153 nm and polydispersity of 0.35 also reported by Bashlouei et al [5] for *Heracleum persicum* essential oil nanoemulsion.

**TABLE 1:** Droplet size and Polydispersity index (PDI) of produced nanoemulsions

Types of nanoemulsions	Droplet size (nm)	Polydispersity index (PDI)
Nanoemulsions containing 1% AEO	75 ± 1.9^a^	0.17 ± 0.03^A^
Nanoemulsions containing 5% AEO	82 ± 2.0^b^	0.18 ± 0.04^A^
Nanoemulsions containing 10% AEO	85 ± 2.2^b^	0.22 ± 0.04^A^
Nanoemulsions containing 15% AEO	95 ± 2.1^c^	0.25 ± 0.05^A^

*Note*: Similar letters show no significant difference in (*α* = 0.05).

Hassanzadeh et al [17] fabricated the nanoemulsions containing garlic essential oil and explained that the droplet size has increased with the increase of the dispersed phase proportion in nanoemulsion formulation. They acclaimed that the small changes in droplet size for all formed nanoemulsions is due to the fact that there was an equal content of essential oil of garlic and surfactant in nanoemulsion formulation and the surfactant molecules were able to surround the encapsulated material.

### 3.2 Antioxidant capacity

**FIGURE 1:**
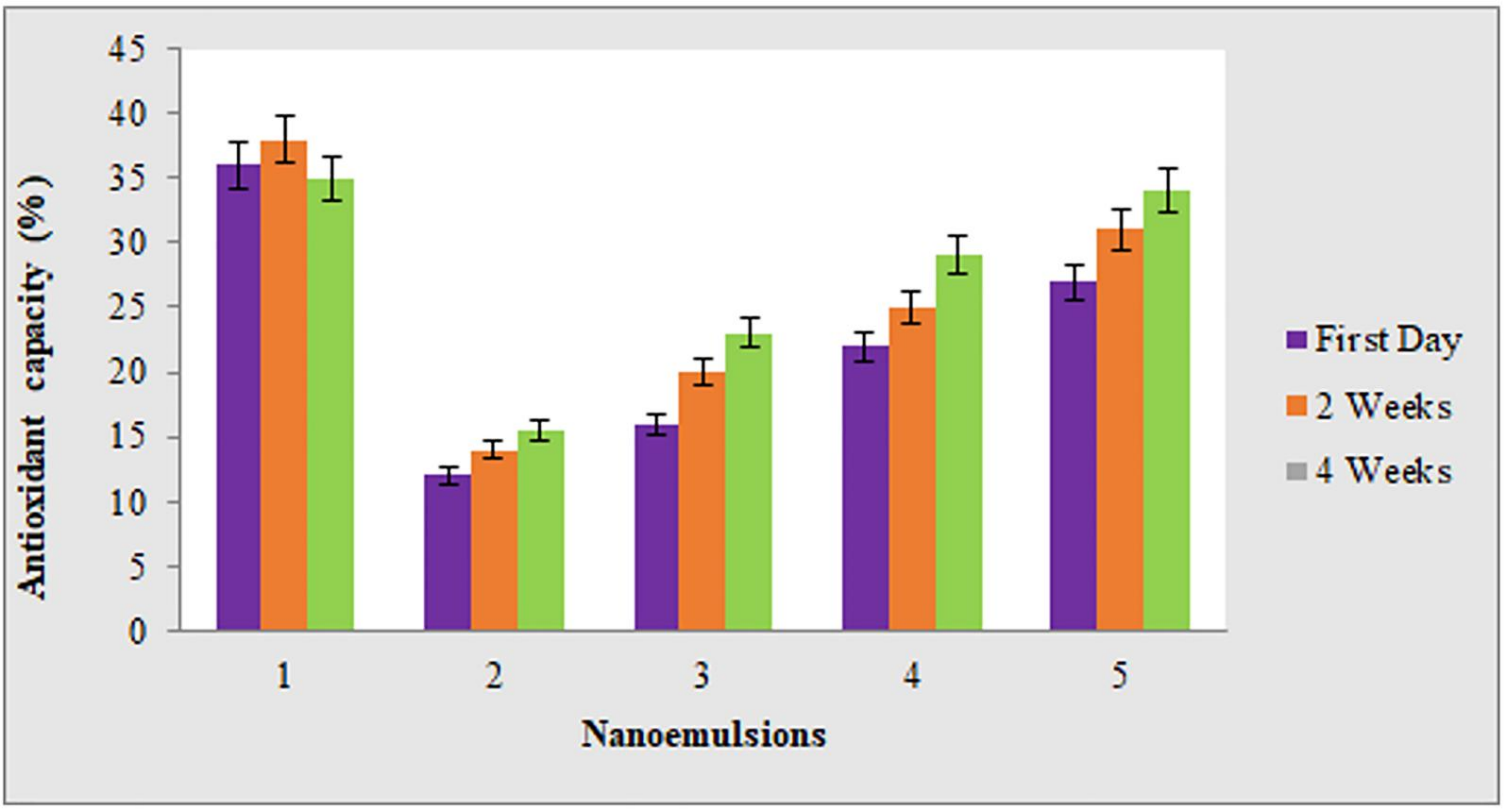
Antioxidant activity of formulated nanoemulsions with concentration of (1 mg/ml) at various interval times. 1: pure angelica essential oil (AEO), 2: nanoemulsion containing 1% AEO, 3: nanoemulsion containing 5% AEO, 4: nanoemulsion containing 10% AEO, 5: nanoemulsion containing 15% AEO.

The antioxidant results by DPPH method showed that the antioxidant capacity was promoted by increasing the percentage of a AEO in the formulation of nanoemulsions and also over time for all nanoemulsions during 2 months storage (*p* < 0.05). As shown in Figure 1, In general, the pure essential oil sample has a higher antioxidant capacity than the nanoemulsion samples, the reason of which can be related to the presence of lower amounts of essential oil in the nanoemulsion samples. But by comparing the antioxidant capacity of nanoemulsion samples during the storage time, it can be seen that the addition of essential oil concentration in the nanoemulsion formulation due to more active ingredient and also the storage time may have caused a significant increase in antioxidant capacity due to the release of effective compounds.

At the similar attempt, Bashlouei et al [5] concluded that *H*. *Persicum* Essential Oil Nanoemulsion could considered as a promising antioxidant and anticancer natural drug in breast cancer therapy. They explained that such antioxidant properties were mainly attributed to (E)‐anethole present in *H*. *persicum* essential oil.

Firuzi et al [18] studied on the composition of essential oils from aerial parts of *Heracleum persicum*, a widely used medicinal plant, and they reported that the *H*. *persicum* showed good antioxidant capacity, which could probably be in part due to the presence of (*E*)‐anethole.

Kenari et al [19] also reported an increase in antioxidant capacity with increasing in concentration of *Heracleum persicum* extract encapsulated by sage (*Salvia macrosiphon*) seed gum and chitosan. By comparing their research and the present study, it can be seen that, in general, *Heracleum persicum* extract has stronger antioxidant properties than its essential oil, which is due to the presence of polyphenols in the extract, while essential oil compounds are mostly related to terpene compounds.

Ha et al. [20] evaluated the antioxidant activity and bioavailability of nanoemulsions formulated with tomato extract enriched with lycopene and they reported that nanoemulsions with particle sizes between 100 and 200 nm showed the highest antioxidant activity. The high antioxidant activity of lycopene nanoemulsions was associated with particle size.

### 3.3 Microbial test

Microbial results obtained by disc diffusion method for two bacteria *Escherichia coli* and *Staphylococcus aureus* treated with pure AEO and nanoemulsions containing different percentages of this essential oil showed that pure AEO had a strong inhibitory effect on both studied bacteria (Table 2). As can be observed in Table 2, nanoemulsion samples containing AEO have a lower inhibitory effect than pure essential oil, which is due to the presence of a lower percentage of essential oil used in the formulation of these samples. Also, among the nanoemulsion samples, with the increase in the percentage of essential oil (as a dispersed phase) in the formulation of nanoemulsions, their inhibitory effect on both bacteria increases significantly. Finally, it can be stated that by comparing the effect of all the samples on the two studied bacteria, it can be found that AEO and its nanoemulsions had a stronger effect on the gram‐positive bacteria (*Staphylococcus aureus*) than on the gram‐negative bacteria (*Escherichia coli*).

**TABLE 2:** Diameter of inhibitory growth zone of E. Coli and S. aurues (mm) treated with nanoemulsions containing essential oils

Types of nanoemulsions	Inhibition zone (mm)	
	*Escherichia coli*	*Staphylococcus aureus*
Pure essential oil (*Heracleum persicum)*	23.4 ± 0.6^a^	31.5 ± 1.6^A^
Nanoemulsions containing 1% essential oil	2.0 ± 0.07^b^	3.5 ± 0.4^B^
Nanoemulsions containing 5% essential oil	5.5 ± 0.3^c^	7.1 ± 1.2^C^
Nanoemulsions containing 10% essential oil	10.1 ± 0.5^d^	13.4 ± 1.3^D^
Nanoemulsions containing 15% essential oil	13.7 ± 0.6^e^	15.5 ± 1.5^E^

*Note*: Different letters show the significant difference in (*α* = 0.05).

Similar results were reported for AEO by Shariatifar et al. [21]. These researchers investigated the antimicrobial properties of this essential oil (by two methods including disc diffusion and broth micro‐dilution) on some of the foodborne pathogens and confirmed the stronger effect of this essential oil on Gram‐positive bacteria.

Ehsani et al. [22] evaluated the antibacterial activity and sensory properties of *Heracleum persicum* essential oil, nisin, and *Lactobacillus acidophilus* against *Listeria monocytogenes* in cheese and confirmed the antimicrobial effect of AEO on this bacterium. Other evidence including antimicrobial effects of *H*. *persicum* EO against *Escherichia* coli and *Campylobacter jejuni* by agar disc diffusion and microdilution assays confirmed these results [23].

In confirmation of the microbial results of this research for nanoemulsions, Hassanzadeh et al. [17] investigated the antimicrobial effect of nanoemulsions containing garlic essential oil using the disk diffusion method and stated that by increasing the concentration of garlic essential oil in the formulation of nanoemulsions, increases their inhibitory effect on each two types of gram‐positive and gram‐negative bacteria. Also, the stronger effect of these nanoemulsions on Gram‐positive bacteria compared to Gram‐negative bacteria has also been reported by them.

### 3.4 Dairy dessert

#### 3.4.1 Dry matter and pH of the desserts

The results related to the measurement of pH and dry matter of dairy desserts enriched with nanoemulsion containing AEO show that, in general, adding nanoemulsion to dairy desserts has caused a decrease in dry matter and a slight increase in pH, while between the samples of dairy dessert enriched with nanoemulsion containing different percentages of AEO has no significant difference. This can be due to the fact that most of the nanoemulsions consist of distilled water and a small percentage of it is essential oil, and generally the nanoemulsions have little dry matter with a slightly higher pH (Table 3).

**TABLE 3:** PH and dry matter of produced dairy dessert samples (enriched and control)

Types of dairy dessert	pH	Dry matter
Control	6.55 ± 0.01^a^	20.5 ± 0.7^A^
Free essential oil^a^	6.56 ± 0.01^a^	20.3 ± 0.5^A^
Nanoemolsions 1% AEO	6.57 ± 0.01^b^	18.6 ± 0.3^B^
Nanoemolsions 5% AEO	6.57 ± 0.02^b^	18.7 ± 0.2^B^
Nanoemolsions 10% AEO	6.58 ± 0.02^b^	18.7 ± 0.2^B^
Nanoemolsions containing 15% AEO	6.58 ± 0.02^b^	18.8 ± 0.2^B^

*Note*: Different letters show the significant difference in (*α* = 0.05).

^a^ Equivalent to 1% nanoemulsion in terms of the amount of pure essential oil.

Other researchers, including Majzoobi et al. [9] have measured the pH and dry matter of dairy dessert enriched with wheat germ powder and found values of 6.52% and 21.57% respectively, as the pH and dry matter of the control sample which are consistent with the results obtained in this research.

#### 3.4.2 Released water from the desserts (synaeresis)

The statistical analysis of the data related to the synaeresis of the dairy dessert samples shows that, in general, the addition of nanoemulsion to the dairy desserts has caused a slight increase in the synaeresis, which seems to be due to the high content water in the nanoemulsion formulation, which causes a decrease in dry matter and the concentration of the thickener in the dessert have and thus increased the synaeresis.

However, by comparing the amount of synaeresis of dairy desserts enriched with nanoemulsion, it can be observed that with the increase in the concentration of essential oil in the formulation of nanoemulsion, especially in higher amounts (10% and 15%), the amount of synaeresis has decreased, which is probably due to the higher viscosity of these nanoemulsions and the more amount of surfactant because the amount of essential oil and the amount of surfactant have increased with a fixed ratio (1:1).

**FIGURE 2:**
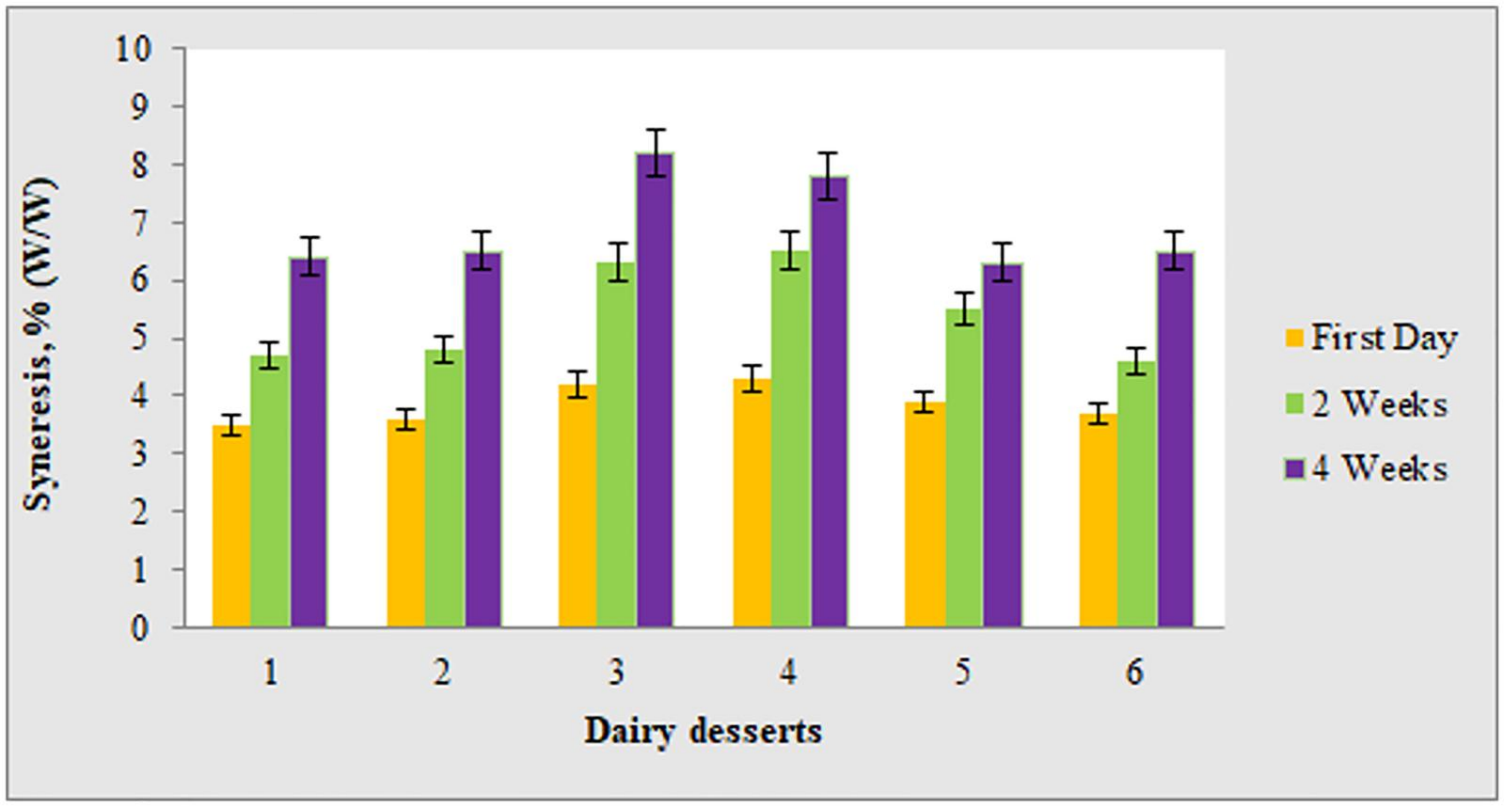
Synaeresis % (W/W) of formulated dairy dessert at various interval times. 1: Control dessert, 2: Dairy dessert enriched with pure essential oil, 3: Dessert enriched with nanoemolsions containing 1% angelica essential oil (AEO), 4: Dessert enriched with nanoemolsions containing 5% AEO, 5: Dessert enriched with nanoemolsions containing 10% AEO, 6: Dessert enriched with nanoemolsions containing 15% AEO.

As shown in Figure 2, The amount of synaeresis for all dairy dessert samples has increased significantly during 4 weeks of storage in the refrigerator, which is consistent with the results of Furlán et al. [24]. These researchers, who have measured the amount of synaeresis during 2 weeks of storage for sugar‐free reduced fat dairy dessert, attributed this increase in synaeresis to the reorganization of starch molecules or retrogradation during cold storage of starch‐based systems.

Also, Maqamikia et al [25] evaluated the apparent viscosity and synaeresis of dairy dessert enriched of vitamin D3‐loaded nanoniosomes produced by different surfactant and explained that changes in the amount of synaeresis were significantly dependent on the percentage of nano‐niosomes.

#### 3.4.3 Microbial characteristics

**FIGURE 3:**
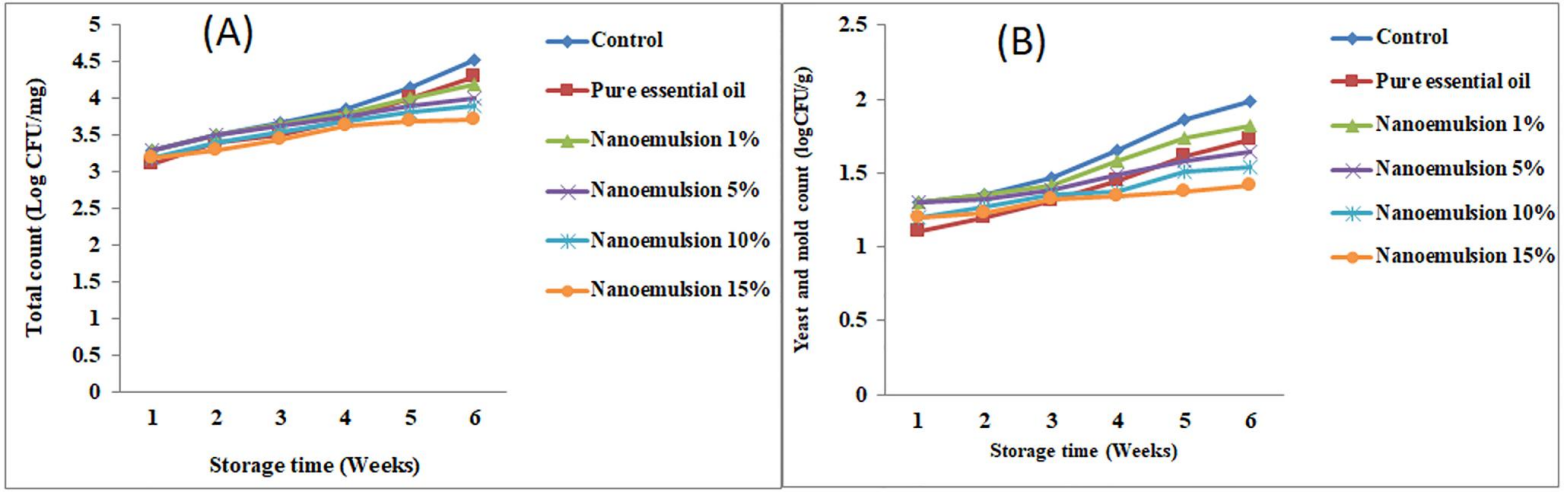
Changes in standard plate count (log CFU/g) (a) and yeast and mould count (log CFU/g) (b) of produced dessert and stored at 4 ± 1°C.

The results of the total count as well as the mould and yeast count related to the dairy dessert samples produced showed that for both tests (total count and mould and yeast), the control samples had more counts than all the enriched samples. Figure 3). Among the enriched dessert samples, the dessert sample enriched with pure essential oil has a different trend, so that at the beginning of the storage period, it has a significant effect on reducing the total count as well as mould and yeast, but over time, its effect has recorded a decreasing trend.

Examining the changes in total count and mould and yeast counts for dairy dessert samples enriched with nanoemulsions showed that these samples had a more stable trend, in other words, at the beginning of the storage period, it had a mild effect on the number of microbes, but it maintained its effect during storage and even showed a stronger effect in high concentrations of essential oil. By comparing the enriched dessert sample containing pure essential oil and the nanoemulsion sample (1% essential oil) with the same percentage of essential oil, it can be observed that nanoemulsions had a slower effect than pure essential oil, but had more stable effects, which is related to the encapsulation of essential oil in nanoemulsions and release of volatile compounds of essential oil during the storage time. In contrast, other researcher reported the no statistically significant difference between the Minimum inhibitory concentration of the free and nanoemulsion extract on *Staphylococcus aureus* [26]. Also, it can be seen from Figure 3a,b that nanoemulsions with higher concentration of essential oil showed their antimicrobial effects earlier and had a stronger antimicrobial effect and reduced the total count and the count of mould and yeast more strongly. In confirmation of the same issue, Hassanzadeh et al. [12] have confirmed the release of more volatile components of garlic essential oil in nanoemulsions with a higher concentration of essential oil.

In the similar cases, Prasad et al. [27] evaluated the effects of packaging materials and essential oils on the storage stability of a dairy dessert and founded that that yeast and mould count was significantly (*p* < 0.05) lowered by essential oil incorporation in the packaging materials. Also, Kalhor and Abdolmaleki [26] produced a dairy dessert based on camel milk enriched with the Echinophora platyloba extract nanoemulsions and *Lactobacillus Plantarum* bacteria by sodium alginate and reported that the bacteria did not grow against the microencapsulated extract.

#### 3.4.4 Rheological analysis

**FIGURE 4:**
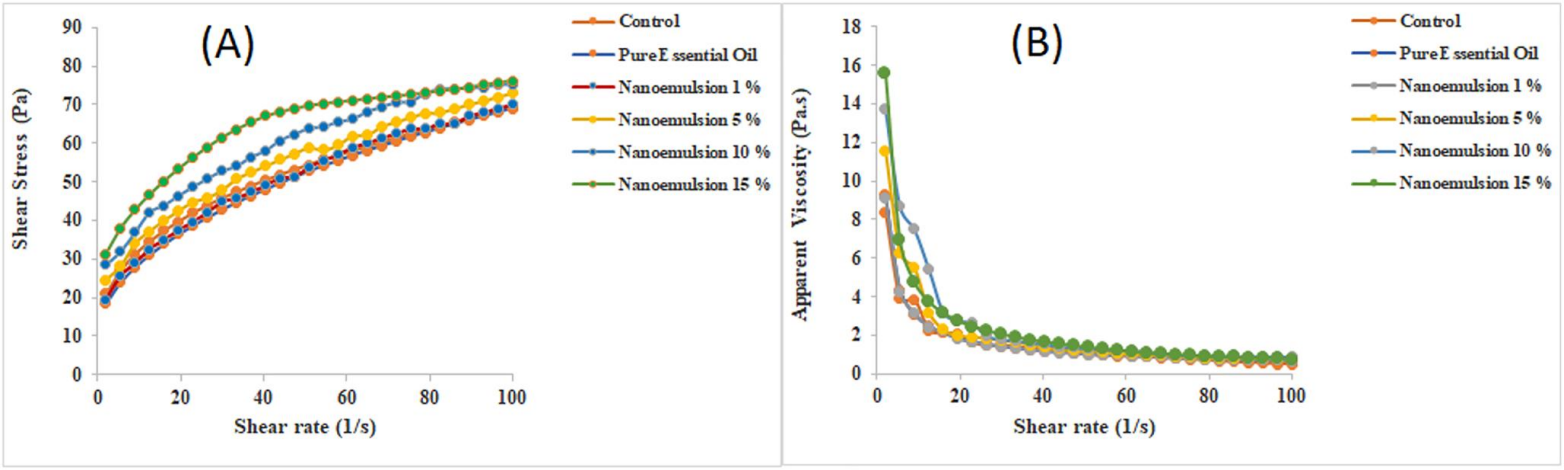
Shear stress (a) and apparent viscosity (b) versus shear rate for the enriched dessert samples.

The relationship between shear stress and shear rate is nonlinear, so dairy dessert samples are classified as non‐Newtonian fluids (Figure 4a). Also, due to the decrease in apparent viscosity with increasing shear rate, dessert samples have a shear thinning (Pseudoplastic) behaviour. The relationship between the apparent viscosity and shear rate of dessert samples is shown in Figure 4b. As the shear rate increased, the apparent viscosity of the samples decreased and they showed a thinning behaviour with the shear.

As shown in Table 4, the dairy dessert sample containing nanoemulsion showed higher apparent viscosity than the one containing free essential oil and control, in the range of applied shear rates.

**TABLE 4:** Power‐law model parameters and apparent viscosity for dairy dessert samples

Samples	n	(Pa.s) K	*R* ^2^	Apparent viscosity at shear rate 50 s^−1^ (Pa.s)
Control	0.314	8.4	0.975	1.00
Free essential oil^a^	0.272	9.26	0.987	1.03
Nanoemolsions 1% AEO	0.237	9.16	0.982	1.02
Nanoemolsions 5% AEO	0.316	11.56	0.996	1.20
Nanoemolsions 10% AEO	0.355	13.7	0.993	1.32
Nanoemolsions 15% AEO	0.321	15.6	0.988	1.37

^a^ Equivalent to 1% nanoemulsion in terms of the amount of pure essential oil.

Apparent viscosity data at intermediate shear rates (20–80 s^−1^) are used to study sensory evaluation and mouthfeel of fluid food materials. For this purpose, the apparent viscosity of the samples at 50 s^−1^ shear rate was compared (Table 4). The results showed that the treatment containing nanoemulsion 15% essential oil had the highest apparent viscosity. The control sample, sample with essential oil and nanoemulsion 1% had the lowest values, meanwhile viscosity of the sample containing higher content of essential oil recorded the higher values of apparent viscosity which this may be due to the higher intrinsic viscosity of the nanoemulsions with a high concentration of essential oil and surfactant.

#### 3.4.5 Dynamic (oscillatory) rheological behaviour

**FIGURE 5:**
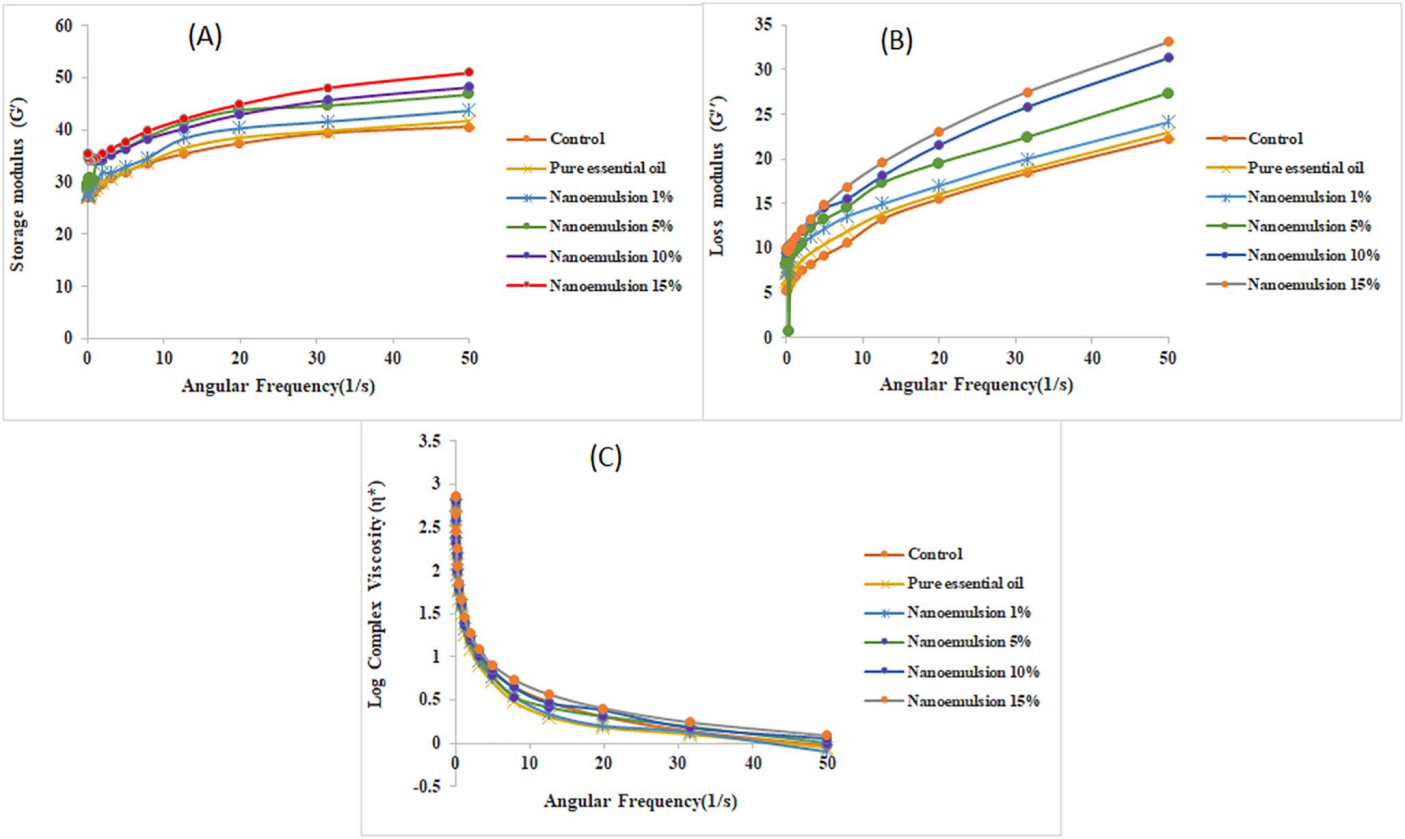
The frequency sweep test of the dairy dessert samples. Storage modulus (a), loss modulus (b) and complex viscosity (c).

A gradual increase in storage modulus and loss modulus of treatments was observed with increasing frequency in the frequency sweep test (Figure 5a,b). The storage modulus indicates the amount of elastic behaviour and the amount of energy recovered per unit volume per complete cycle of the strain wave, and the loss modulus or viscosity modulus (G'') indicates the amount of flow behaviour and the amount of energy lost per unit volume per complete cycle of strain wave. In the frequency sweep test, if G'' < G′, the sample shows solid viscoelastic behaviour and if G'' > G′, the sample shows liquid viscoelastic one. In this research, the storage modulus data were higher than loss modulus in all frequencies which shows the characteristic of viscoelastic materials behaviour such as dispersions and gels [28]. The elastic response dominates the viscous one, for which it may be related to the structuring of molecules of the particular custard system, leading to this gel response [29]. A dependence on frequency of both moduli is observed, as well as a function of the types of enrichment in the gel structure. In all three parts of Figure 5a–c, higher values are observed for samples containing nanoemulsion and especially in higher concentrations of essential oil.

Many researchers have reported values of the viscoelastic parameters at frequency of 0.5–1 Hz, which indicates a value in which a human mouth begins to make structural changes. At that frequency of 0.5 Hz, the viscoelastic parameters for the enriched dessert samples are shown in Table 5. As can be observed in Table 5, the magnitudes for both moduli, the most consistent or firmer samples were the desserts enriched with nanoemulsion, with the highest moduli (Gʹ, G" and complex modulus G*).

**TABLE 5:** Values of the viscoelastic parameters for enriched and control desserts at a frequency of 0.5 Hz

Samples	G'	G"	Tan *δ*	G*
Control	27.5	6.03	0.219	28.15
Free essential oil^a^	27.8	7.3	0.262	28.74
Nanoemolsions 1% AEO	28.8	9.3	0.313	30.18
Nanoemolsions 5% AEO	30.8	9.03	0.293	32.09
Nanoemolsions 10% AEO	34.4	10.4	0.302	35.93
Nanoemolsions 15% AEO	34	10.2	0.3	35.49

^a^ Equivalent to 1% nanoemulsion in terms of the amount of pure essential oil.

The ratio of G'' to G′ indicates another parameter called the loss tangent (Tan δ). If the loss tangent is greater than 1, it means that the viscoelastic material is liquid, and if it is less than 1, it indicates the solid viscoelastic behaviour in the material. The results showed that in all dessert samples tan *δ* was less than one, which indicates the higher elastic property than viscose one and consequently, the solid viscoelastic behaviour of the samples.

Other researchers also reported these viscoelastic parameters for different dairy desserts, some of which can be mentioned. Alamprese and Mariotti [30] evaluated the viscoelastic behaviour of different puddings after storage at 4°C for one day, and reported the values of 105–442 Pa for Gʹ, 1.73–68.5 Pa for G", and 12.6–445 Pa for G*. Also, Torres et al. [31] showed values at 1 Hz of the same magnitude, for dairy dessert samples with and without inulin through storage time, and they reported an increase in these dynamic moduli. Zapata‐Noreña et al. [32] also reported a range of 0–550 Pa for Gʹ and 0–100 Pa for G" for skimmed and whole milk custard desserts at 1 Hz, which are comparable to enriched and control dairy dessert in the present study.

#### 3.4.6 Sensory evaluation

**FIGURE 6:**
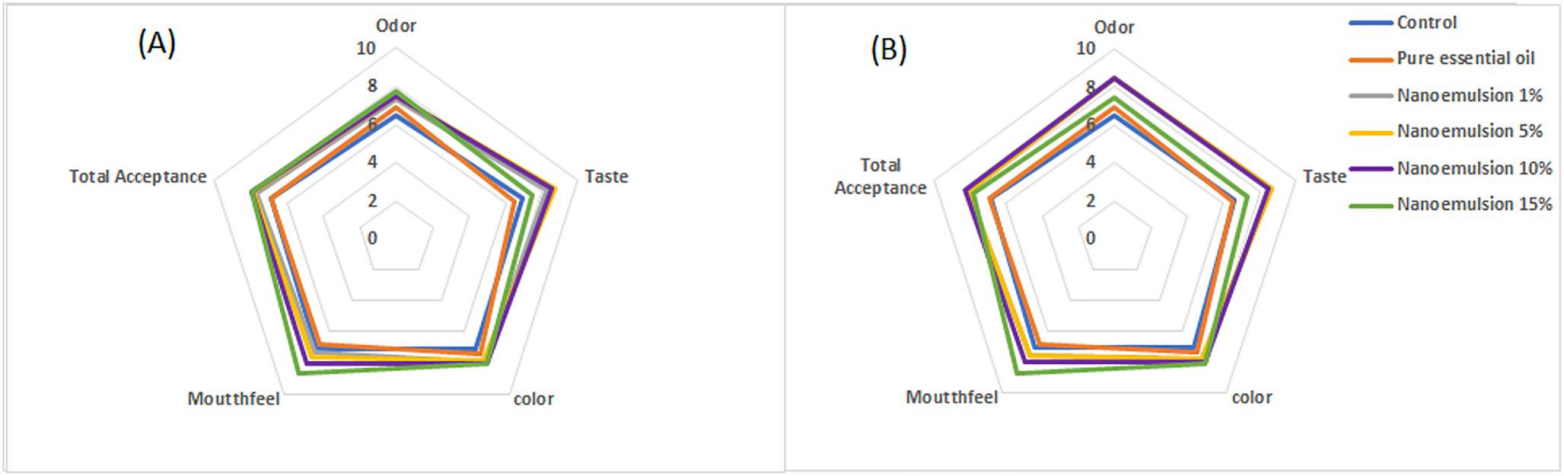
Spider plot for describing the sensory properties of enriched and control dairy dessert for first day (a) and after 30 days (b) and stored at 4 ± 1°C.

Sensory analysis including taste, colour, odour, mouthfeel and total acceptance showed that samples containing AEO especially in higher concentration of essential oil had highest scores in all organoleptic characteristics. In general, samples containing pure essential oil had the lowest scores in all organoleptic characteristics (Figure 6). By comparing Figure 6a,b, it can be concluded that despite the superiority of samples containing nanoemulsion for all indicators on the first day and after 30 days of storage, the samples containing nanoemulsion with a lower percentage (10% and 5%) on the 30th day of grades have obtained higher score than the first day, which can be due to the slow release of essential oil volatiles during storage. Also, the samples containing pure essential oil in both times of the test (first day and 30th day) and especially on the first day due to the sudden release of essential oil volatile substances, caused an unpleasant taste in the product and significantly reduced the sensory evaluation scores. Regarding the effect of nanoemulsions containing plant essential oils on the sensory properties of food products, it can be referred to the research of Mansouri et al. [33] which investigated the stability and antibacterial activity of *Thymus daenensis* L. essential oil nanoemulsion in mayonnaise. Their optimal nanoemulsion achieved significantly higher sensory scores (taste, appearance, and mouthfeel) than the pure essential oil in mayonnaise. Also, the most acceptability achieved from the panelist for ice‐cream sample enriched by Nigella sativa oil nanoemulsion (5%) compared to the other samples [34].

At a similar attempt, Flamminii et al [35] measured the sensory properties of mayonnaise enriched with encapsulated olive leaf phenolic extracts and reported that the enriched mayonnaise samples, in particular the system fortified with OLE‐loaded microparticles, displayed the lowest total acceptability from the panelists. Also, the enriched samples showed a lower spreadability and a higher salty and bitter perception, leading to reduced total acceptability.

## 4 CONCLUSION

The ever‐increasing interest in the use of essential oils as natural antimicrobials and preservatives in the food industry has been driven in the last years by the growing consumers' demand for natural products with improved microbial safety, and fresh‐like organoleptic properties. Nanoemulsions efficiently contribute to support the use of essential oils in foods by increasing their dispersibility in the food areas where microorganisms grow and proliferate, by reducing the impact on the quality attributes of the product, as well as by enhancing their antimicrobial activity. Choosing the type of essential oil for food enrichment is not only related to its nutritional, antimicrobial and antioxidant properties, but also depends on the type of food selected in terms of colour, taste and product quality, marketability and compatibility of essential oil with food. In the present study, the increase in shelf life, the improvement of sensory and rheological characteristics of dairy desserts enriched with nanoemulsions compared to control dairy dessert samples and samples enriched with pure essential oil have been observed, which shows the importance of using nanoencapsulation to protect and release control of bioactive compounds including plant essential oils and it can encourage more researchers in this direction.

## AUTHOR CONTRIBUTIONS

**Narin Mhemmedamin Nanakali**: Investigation; Methodology, Supervision; Writing – original draft Formal analysis and Writing – review & editing.

## CONFLICT OF INTEREST

Author declare that they have no conflict of interest.

## Data Availability

The data that support the findings of this study are available on request from the corresponding author. The data are not publicly available due to privacy or ethical restrictions.
